# Anti‐Viral Treatment Eligibility and Treatment Uptake Among People With Chronic Hepatitis B in Australia: The REACH‐B Study

**DOI:** 10.1111/jvh.70238

**Published:** 2026-11-02

**Authors:** Behzad Hajarizadeh, Renjy Nelson, Jacob George, Josh Hanson, Jane Davies, Marianne Martinello, Tracey Brown, Ian Wong, Tasnim Hasan, Lester Mascarenhas, James O'Beirne, Amanda Wade, Marilou Capati, Georgina Trotter, Sarah Thomas, Lloyd Nash, Benjamin Cowie, Nicole Allard, Thomas Tu, Lien Tran, Stephanie Howard, Robert Monaghan, Jessica Carter, Elise Tu, Amanda Erratt, Joanne Carson, Gregory J. Dore, Gail V. Matthews, the REACH‐B Study Group

**Affiliations:** ^1^ The Kirby Institute University of New South Wales (UNSW) Sydney New South Wales Australia; ^2^ The Queen Elizabeth Hospital and Royal Adelaide Hospital Adelaide South Australia Australia; ^3^ Storr Liver Centre, Westmead Hospital, and The Westmead Institute for Medical Research University of Sydney Sydney New South Wales Australia; ^4^ Cairns and Hinterland Hospital and Health Service, and Torres and Cape Hospital and Health Service Cairns Queensland Australia; ^5^ Menzies School of Health Research Darwin Northern Territory Australia; ^6^ Justice Health and Forensic Mental Health Network NSW Sydney New South Wales Australia; ^7^ Blacktown Mt Druitt Hospital Sydney New South Wales Australia; ^8^ Utopia Refugee and Asylum Seeker Health Melbourne Victoria Australia; ^9^ Sunshine Coast University Hospital Sunshine Coast Queensland Australia; ^10^ Barwon Health Geelong Victoria Australia; ^11^ Top End Medical Centre Darwin Northern Territory Australia; ^12^ Western Australia Country Health Service Perth Western Australia Australia; ^13^ Royal Melbourne Hospital Melbourne Victoria Australia; ^14^ WHO Collaborating Centre for Viral Hepatitis, and the Peter Doherty Institute for Infection and Immunity Melbourne Victoria Australia; ^15^ Cohealth Melbourne Victoria Australia; ^16^ Hepatitis B Voices Australia Melbourne Victoria Australia; ^17^ St Vincent's Hospital Sydney New South Wales Australia

**Keywords:** antiviral therapy, cirrhosis, clinical guidelines, cohort, HBV

## Abstract

Treatment indication for chronic hepatitis B (CHB) varies across clinical guidelines. This study evaluated treatment eligibility and uptake in a large Australian cohort of people with CHB. The REACH‐B study recruited people with CHB managed through a national network of healthcare services. Participants were categorised into three groups: (1) receiving antiviral therapy; (2) untreated despite eligibility; (3) untreated and ineligible. Treatment eligibility was determined using Australian Pharmaceutical Benefits Scheme (PBS) criteria, and by international clinical guidelines. Among 3648 participants (56% male; median age 48 years; 49% born in North‐East or South‐East Asia; 27% First Nations Australians), 14% were HBeAg positive and 7% had cirrhosis. Overall, 47% (*n* = 1666) were on antiviral therapy, 45% (*n* = 1573) were untreated and ineligible and 7% (*n* = 251) were untreated despite eligibility based on Australian PBS criteria. In adjusted analysis, the likelihood of remaining untreated despite eligibility was higher among women (aOR 1.59, 95% CI 1.34, 1.88) and younger participants (age 41–60 vs. > 60 years: aOR 1.51, 95% CI 1.01–2.28; ≤ 40 vs. > 60 years: aOR 2.65, 95% CI 1.60, 4.40). The proportion untreated despite eligibility increased to 13% when applying 2024 World Health Organisation guidelines and ranged 2%–44% based on other guidelines, highlighting the variability across the guidelines. In conclusion, among REACH‐B participants, most eligible individuals receive treatment. Lower treatment uptake among women and younger people despite eligibility partly reflects lower threshold for elevated ALT in women, and clinician determination of non‐CHB related ALT elevation. As treatment guidelines evolve and eligibility criteria broaden, monitoring treatment patterns is important.

## 1 Introduction

Hepatitis B virus (HBV) infection is a global public health concern, with an estimated 258 million people living with chronic HBV infection [1] and 1.1 million deaths attributed to HBV [2] in 2022. Although current HBV antiviral therapies are highly effective in suppressing the virus, clinical guidelines do not recommend therapy for everyone. HBV treatment is indicated for specific patient groups, based on liver disease stage, risk of progression or hepatocellular carcinoma (HCC), or prevention of mother‐to‐child transmission or reactivation during immunosupression [3, 4, 5, 6, 7]. For those not indicated for treatment, regular monitoring is recommended, generally every 6–12 months, with assessment of virological and liver disease markers and HCC surveillance for at‐risk individuals.

Specific indications for HBV antiviral therapy (also called treatment eligibility) are based on a complex algorithm incorporating several clinical factors, including liver disease stage, HBV viral markers, liver function tests, co‐morbidities, as well as individual characteristics such as age and family history of advanced liver disease [3, 4, 5, 6, 7]. There is no universal consensus on treatment eligibility, with variance across guidelines [3, 4, 5, 6, 7]. Further, several guidelines have recently broadened to expand indications for treatment, although the degree to which eligibility has been extended varies [3, 7, 8]. Even when considering narrower treatment eligibility criteria, many individuals do not receive guideline‐based care [9, 10, 11]. Reasons for this, in addition to undiagnosed HBV infection, include clinicians not following guideline recommendations [12, 13], limited access to necessary services and tests such as HBV DNA quantification (particularly in resource‐limited settings) [14, 15], limited culturally competent services [16] and individual choice [17].

The revisions to global treatment guidelines, including the 2024 World Health Organization (WHO) HBV clinical guidance, which recommend simplified criteria for initiating antiviral therapy, have been estimated to result in a considerably larger potential pool of individuals eligible for treatment [3]. However, there are limited data on how these revised criteria will change the proportion of people eligible for treatment in specific settings compared to current models [18, 19, 20, 21].

In Australia, an estimated 219,800 individuals were living with chronic HBV infection in 2023, with the majority born overseas or identifying as Aboriginal and/or Torres Strait Islander Australians [22, 23], hereafter respectfully referred to collectively as First Nations Australians. Subsidised HBV antiviral therapy is available in Australia for patients who meet treatment criteria specified by the Pharmaceutical Benefit Scheme (PBS; Supplementary Table 1), a federal government program that subsidises prescription medications [24]. The PBS criteria largely follow the Australian clinical guidelines for HBV developed by a national Consensus process published in 2022 [4]. However, data are lacking on adherence to PBS criteria or clinical guideline recommendations for HBV treatment. Characterising the demographic and clinical profile of people living with chronic HBV infection in Australia and their engagement with clinical care is particularly important given the changing HBV treatment landscape [25].

In this study, we used baseline data from a national cohort study of people living with chronic HBV infection enrolled from a diverse range of clinical services to characterise the demographic and clinical profiles of people with chronic HBV infection in Australia. We also evaluated the proportion eligible for antiviral therapy based on both PBS criteria and several major national and international clinical guidelines and uptake among those eligible for treatment.

## 2 Methods

This study is reported in accordance with the STROBE guideline for reporting cohort studies [26].

### 2.1 Study Design and Setting

The Real‐world Assessment of people living with Chronic Hepatitis B in Australia (REACH‐B Study) is a multi‐centre cohort study of people living with chronic HBV infection. The study setting is a national network of diverse healthcare services, providing HBV clinical care across Australia. The study commenced data collection in February 2022 and by September 2025, the REACH‐B network included 18 active sites (17 sites with available data for this analysis) from six of Australia's eight states and territories, including 15 hospital‐based viral hepatitis specialist services (*n* = 5 have affiliated community outreach services), two primary care clinics and one state‐wide prison‐based health service.

### 2.2 Participants and Study Procedure

Eligible participants included adults (≥ 18 years) diagnosed with chronic HBV infection who attended a REACH‐B study site within 12 months prior to study commencement. Chronic HBV infection is defined as positive HBV surface antigen (HBsAg) persisting for more than 6 months. Consecutive eligible patients were enrolled.

Standard clinical care data was collected from existing medical records at each site. A secure REDCap dataset was developed and hosted by the Coordinating Centre for the collection of site‐level data. However, given the diversity of systems used by sites to record data, the study provided customised options for sites to submit data. Twelve sites entered data manually using a standardised electronic data collection form developed within REDCap, which automatically transferred the data to the central dataset. Six sites used data extraction technology to export data from their existing electronic databases directly into the REDCap dataset. Three of these six sites also supplemented their extracted data with manual data entry as described above.

The collected data include demographic characteristics, past medical history (e.g., diabetes, hepatitis C, HIV), most likely mode of HBV infection, year of HBV diagnosis, family history of HCC, laboratory tests results (e.g., HBV and hepatitis D virus (HDV) serological markers, HBV DNA, liver function tests), liver fibrosis assessments (e.g., transient elastography score, AST to Platelet Ratio Index (APRI), liver biopsy report), HCC screening and HBV clinical management. Follow‐up clinical data are collected from subsequent visits, at a minimum of once per year. The follow‐up data include laboratory tests results, liver fibrosis assessments, HCC screening and HBV clinical management.

### 2.3 Study Outcomes

The primary study outcome was HBV antiviral therapy indication or eligibility (referred to as treatment eligibility hereafter), determined by categorisation into four groups: (1) receiving treatment; (2) not receiving treatment, but eligible for treatment; (3) not receiving treatment and not eligible for treatment; and (4) insufficient data to determine treatment eligibility. Among those not receiving treatment, eligibility was defined based on the PBS criteria in 2024 [24] and recommendations by one national and five international HBV clinical guidelines (most recent versions). These clinical guidelines have been developed by the Gastroenterological Society of Australia (GESA) in 2022 [4], Asian‐Pacific Association for Study of Liver (APASL) in 2015 [6], American Association for Study of Liver Diseases (AASLD) in 2026 [5], European Association for Study of Liver (EASL) in 2025 [7], WHO in 2024 [3] and the Chinese Medical Association (CMA) in 2022 [8]. Definition of treatment eligibility varied across the guidelines and included clinical factors such as alanine transaminase (ALT) level, HBV viral load, HBV e antigen (HBeAg) status, liver fibrosis and co‐morbidities, as well as individual characteristics such as age, sex and family history of cirrhosis or HCC. Guideline‐specific thresholds for ALT elevation (IU/L) were applied, including > 19 for women and > 30 for men (PBS, GESA, WHO, CMA); > 25 for women and > 35 for men (AASLD); and > 40 for both sexes (EASL, APASL) (Supplementary Figure 1). Cirrhosis status was determined using a hierarchical approach. Where available, cirrhosis status as identified by the study site was used. If this information was missing, a transient elastography score cut‐off of 11 kPa was used to indicate cirrhosis status, consistent with the EASL recommendations [7]. In the absence of above data, the APRI was used, with values ≤ 1.0 considered as no cirrhosis and ≥ 2.0 indicative of cirrhosis. Family history of cirrhosis or HCC and co‐morbidities were not considered in treatment eligibility criteria in this study given limited data available for most participants. Recommendations for treating pregnant women or for prophylactic treatment among individuals on immunosuppressive therapy were also not considered.

### 2.4 Statistical Analysis

The baseline data of participants at enrolment was used for all analyses. Participants included in analyses were those with available data on HBV DNA and ALT levels, and clinical management plan. The distribution of demographic and clinical characteristics of participants was evaluated and presented using proportion for categorical variables and median and inter‐quartile range for continuous variables.

For treatment eligibility analyses, participants receiving antiviral therapy and those who were monitored without treatment at enrolment were included. Thus, a small number of participants who were undergoing initial HBV assessment or receiving other forms of HBV management (e.g., prophylactic treatment, follow‐up post HBsAg seroconversion or treatment cessation, clinical trial‐based treatment) were excluded from this analysis. For each participant, treatment eligibility was determined separately using the PBS criteria [24] and each of the six clinical guidelines [3, 4, 5, 6, 7, 8]. After determining treatment eligibility and current clinical management status for each participant, they were categorised into four groups, as described previously.

In subsequent analyses, treatment eligibility according to the PBS criteria was used as the outcome given that PBS criteria define the standard practice for prescribing in Australia. The PBS criteria for HBV prescribing are largely consistent with the national GESA guidelines for HBV treatment, with the main difference being that GESA recognises evidence of liver fibrosis as an alternative to elevated ALT for determining treatment eligibility (Supplementary Figure 1). For these analyses, first, the distribution of treatment eligibility categories was evaluated, stratified by cirrhosis and HBeAg status (as the key factors contributing to the risk of liver fibrosis progression and HCC). Multivariable logistic regression was used to assess whether sex, age, First Nations Australians identity, or country of birth was associated with not receiving HBV antiviral therapy despite eligibility. Clinical factors such as ALT, HBV DNA, HBeAg and cirrhosis were not included in the regression models to avoid overadjustment bias, since they are definitional components of treatment eligibility, as the analysis outcome [27]. Robust variance estimates were used in all models, with study sites specified as the groups (clusters) to adjust for potential correlation of participants within each site.

Statistical significance was assessed at *p* < 0.05 (two‐sided *p*‐values). All analyses were conducted using Stata 17.0 (StataCorp, College Station, TX, US).

## 3 Results

### 3.1 Participant Characteristics

By September 2025, a total of 4342 participants were recruited from 17 sites in six states and territories, of whom 3648 participants had available ALT and HBV DNA at baseline and were included in analyses.

Baseline characteristics of participants have been summarised in Table 1. Overall, 44% of participants were women, the median age was 48 years, 27% identified as First Nations Australians and 68% were born overseas (49% in North‐East or South‐East Asia). Clinical characteristics of participants included 14% HBeAg positive and 7% with cirrhosis. Baseline characteristics varied by study site (Supplementary Table 2).

**TABLE 1:** Demographic and clinical characteristics of participants, overall and by HBV clinical management status at enrolment.

Baseline characteristics	Total^a^ *n* = 3648	Being monitored *n* = 1852	Receiving treatment *n* = 1666
Sex, *n* (%)			
Female	1610 (44)	888 (48)	681 (41)
Male	2032 (56)	963 (52)	981 (59)
Not available	6 (< 1)	1 (< 1)	4 (< 1)
Median age (IQR)	48 (40, 59)	46 (39, 57)	52 (41, 62)
First Nations Australians, *n* (%)			
Yes	1003 (27)^b^	758 (41)	220 (13)
No	2643 (72)	1092 (59)	1446 (87)
Not available	2 (< 1)	2 (> 1)	0
Region of birth, *n* (%)			
Australia	1157 (32)	809 (44)	314 (19)
North‐East or South‐East Asia	1801 (49)	686 (37)	1048 (63)
Sub‐Saharan Africa	214 (6)	115 (6)	87 (5)
Pacific	121 (3)	59 (3)	57 (3)
West or Central Asia	116 (3)	53 (3)	57 (3)
South Asia	88 (2)	56 (3)	30 (2)
Other regions	113 (3)	55 (3)	55 (3)
Not available	38 (1)	19 (1)	18 (1)
Reported mode of HBV, *n* (%)			
Vertical or horizontal via family	2329 (63)	1397 (75)	856 (51)
Injecting drug use or unsafe tattoo	114 (3)	48 (3)	56 (3)
Sexual transmission	53 (1)	21 (1)	27 (2)
Other	53 (1)	26 (1)	25 (1)
Unknown	1099 (30)	360 (19)	702 (42)
HBeAg positive, *n* (%)			
Negative	2843 (78)	1578 (85)	1170 (70)
Positive	499 (14)	147 (8)	329 (20)
Not available	306 (8)	127 (7)	167 (10)
HBV DNA, *n* (%)			
Undetected or < 20 IU/mL	1926 (53)	562 (30)	1331 (80)
20–2000 IU/mL	1124 (31)	909 (49)	167 (10)
2000–20,000 IU/mL	299 (8)	220 (12)	61 (4)
> 20,000 IU/mL	299 (8)	161 (9)	107 (6)
Elevated ALT (F > 19 IU/L, M > 30 IU/L), *n* (%)	2127 (58)	1043 (56)	999 (60)
Elevated ALT (F > 25 IU/L, M > 35 IU/L), *n* (%)	1517 (42)	733 (40)	723 (43)
Elevated ALT (F/M > 40 IU/L), *n* (%)	858 (24)	400 (22)	417 (25)
HDV infection, *n* (%)			
No (HDV Ab or HDV RNA negative)	1222 (34)	554 (30)	607 (36)
Yes (HDV RNA positive)	26 (1)	13 (1)	10 (1)
HDV Ab positive, HDV DNA results not available	37 (1)	20 (1)	17 (1)
HDV Ab and HDV RNA results not available	2363 (65)	1265 (68)	1032 (62)
Cirrhosis, *n* (%)			
No	3299 (90)	1793 (97)	1389 (83)
Yes	255 (7)	28 (2)	222 (13)
Not available	94 (3)	31 (2)	55 (3)
HIV co‐infection, *n* (%)			
No	3154 (86)	1687 (91)	1344 (81)
Yes	44 (1)	7 (< 1)	36 (2)
Not available	450 (13)	158 (9)	286 (17)
HCV status, *n* (%)			
HCV Ab negative	3188 (87)	1664 (90)	1409 (85)
HCV Ab positive, HCV RNA negative	97 (3)	49 (3)	41 (2)
HCV RNA positive	24 (1)	8 (< 1)	14 (1)
Not available	339 (9)	131 (7)	202 (12)

^a^ 130 participants were undergoing initial HBV assessment or receiving other forms of HBV management.

^b^ 649 participants were identified as Aboriginal, 306 participants as Torres Strait Islander, and 48 participants as both.

Among participants, 46% (*n* = 1666) were receiving antiviral therapy, 51% (*n* = 1852) were being monitored (without treatment) and 3% (*n* = 130) were receiving other forms of HBV management (Table 1). Participants' characteristics in the two groups who were receiving treatment or being monitored are summarised in Table 1. More than half of participants (53%) had undetectable or unquantifiable (< 20 IU/mL) HBV DNA, including 80% of those receiving antiviral therapy and 30% of those undergoing monitoring. The proportion of participants with elevated ALT varied considerably (24%–58%) by ALT threshold. Distribution of elevated ALT by HBV DNA level is illustrated in Figure 1. Using an ALT threshold of > 19 IU/L for women and > 30 IU/L for men, the proportion with elevated ALT was 58% and varied by HBV DNA level: 55%–56% among those with HBV DNA ≤ 2000 IU/mL, 63% among those with HBV DNA 2001–20,000 IU/mL and 83% among those with HBV DNA > 20,000 IU/mL. Most participants (66%) did not have available data on HDV test results. Overall, 81 participants (2%) were HDV antibody positive, among whom 23 (28%) were HDV RNA positive, 21 (26%) were HDV RNA negative, while for 36 (44%) no HDV RNA test results were available. Among individuals with available data to identify their HDV status, 2% (*n* = 26/1248) were HDV RNA positive (Table 1).

**FIGURE 1:**
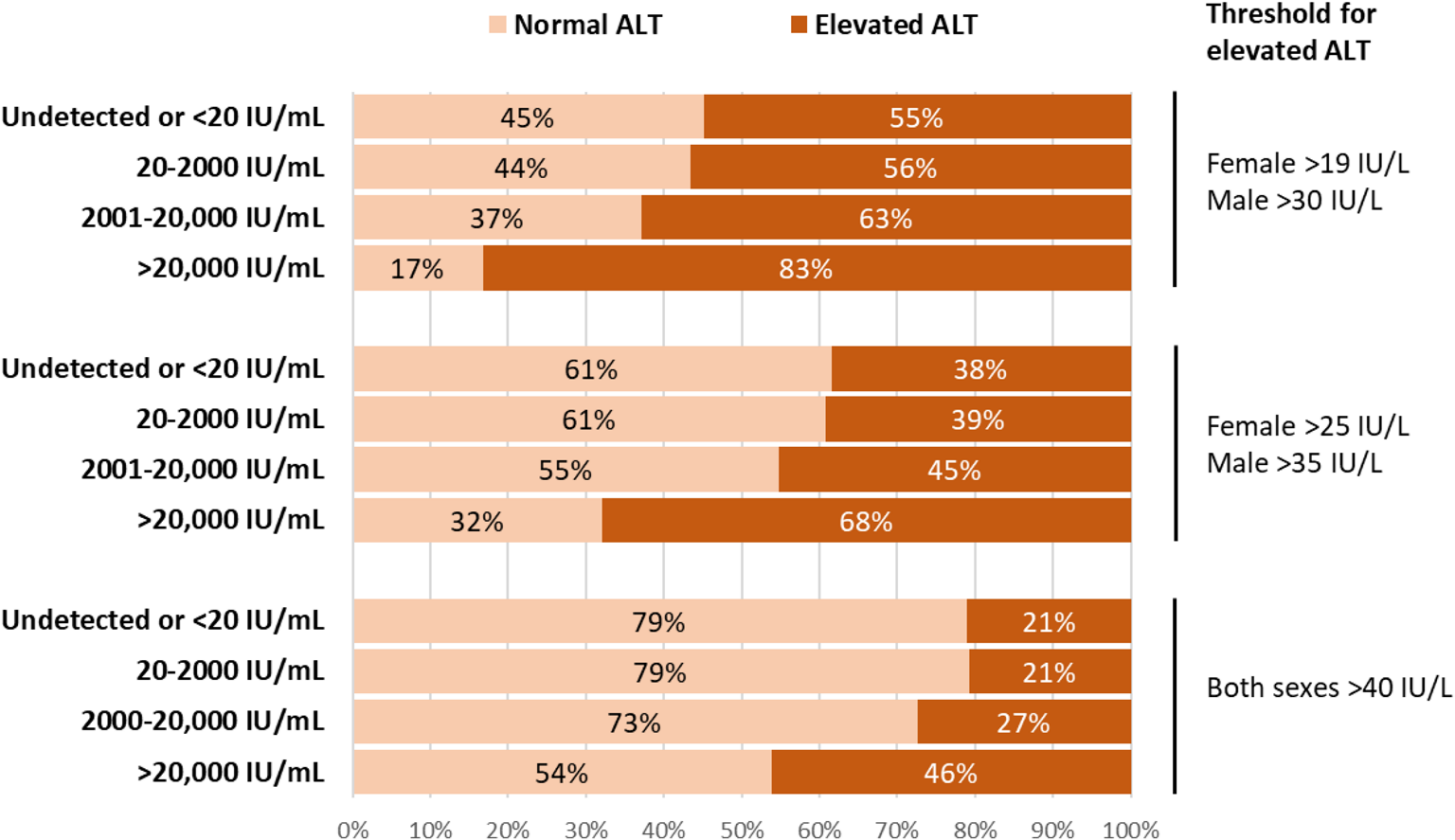
Distribution of elevated ALT by HBV DNA level, using various thresholds.

### 3.2 HBV Antiviral Therapy

Among 1666 participants receiving antiviral therapy, 68% (*n* = 1140) were on entecavir, 26% (*n* = 439) on tenofovir (*n* = 25 tenofovir alafenamide, *n* = 414 tenofovir disoproxil) and 5% (*n* = 87) on other regimens or combination therapy. When stratified by sex and age, differences in the pattern of antiviral therapy regimen use were observed. Among females of peak child‐bearing age (18–44 years), tenofovir was the predominant therapy (63% compared to 35% on entecavir; Figure 2).

**FIGURE 2:**
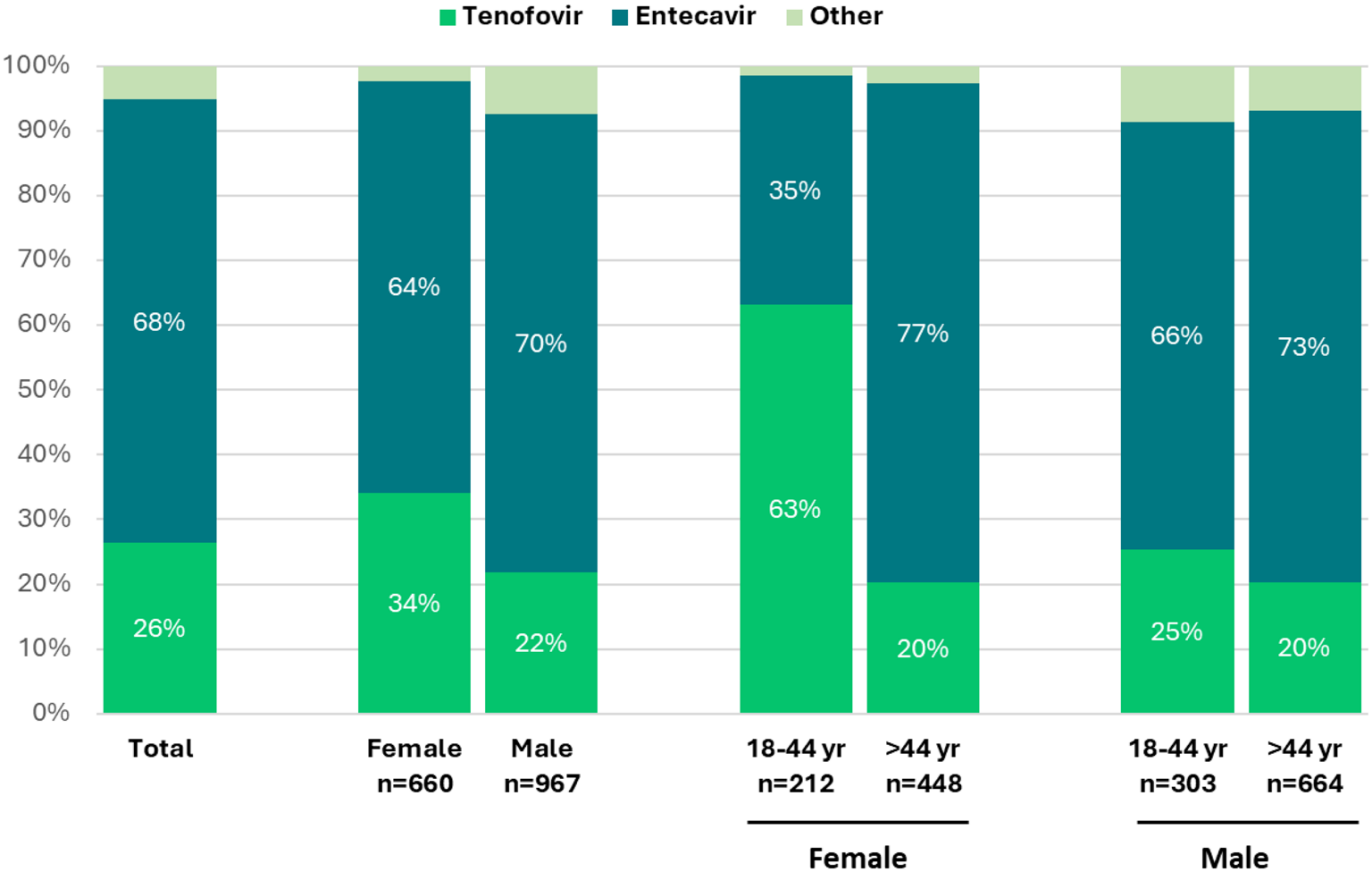
Distribution of HBV antiviral therapy regimens among participants receiving therapy, by sex and age.

### 3.3 Eligibility for Antiviral Therapy

Based on Australian prescribing criteria (PBS), 92% of total participants were either receiving antiviral therapy (47%) or were untreated and ineligible (45%), while 7% were untreated despite eligibility (Figure 3A). Among the subset of participants who were being monitored (*n* = 1140), 14% were eligible for treatment but not receiving it based on the Australian PBS criteria. Applying the Australian Clinical guidelines only increased the proportion eligible by 1%–2%. However, this proportion increased to 25% if WHO guideline criteria were used and ranged from 3% (APASL 2015) to 83% (Chinese Medical Association 2022), applying other international guidelines (Figure 3B).

**FIGURE 3:**
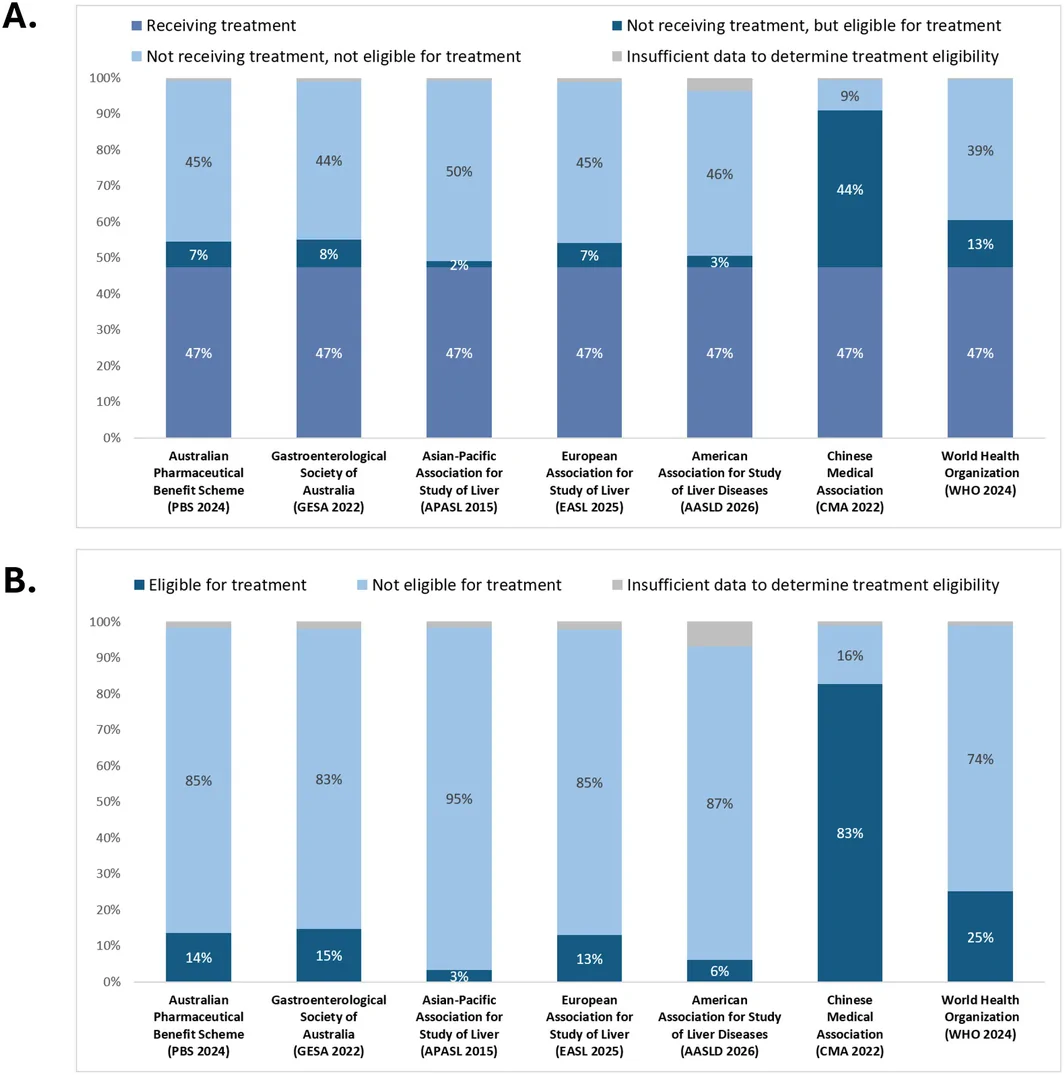
Proportion of participants eligible for HBV antiviral therapy based on the various national and international clinical guidelines among (A) the overall REACH‐B population and (B) participants who were monitored without treatment.

Among 250 individuals with cirrhosis, 89% (*n* = 222) were receiving antiviral therapy, while 9% (*n* = 22) were eligible but not receiving treatment based on the Australian PBS criteria (Figure 4). Among 476 HBeAg positive individuals, 69% (*n* = 329) were receiving antiviral therapy, while 15% (*n* = 70) were eligible but not receiving treatment based on the PBS criteria (Figure 4).

**FIGURE 4:**
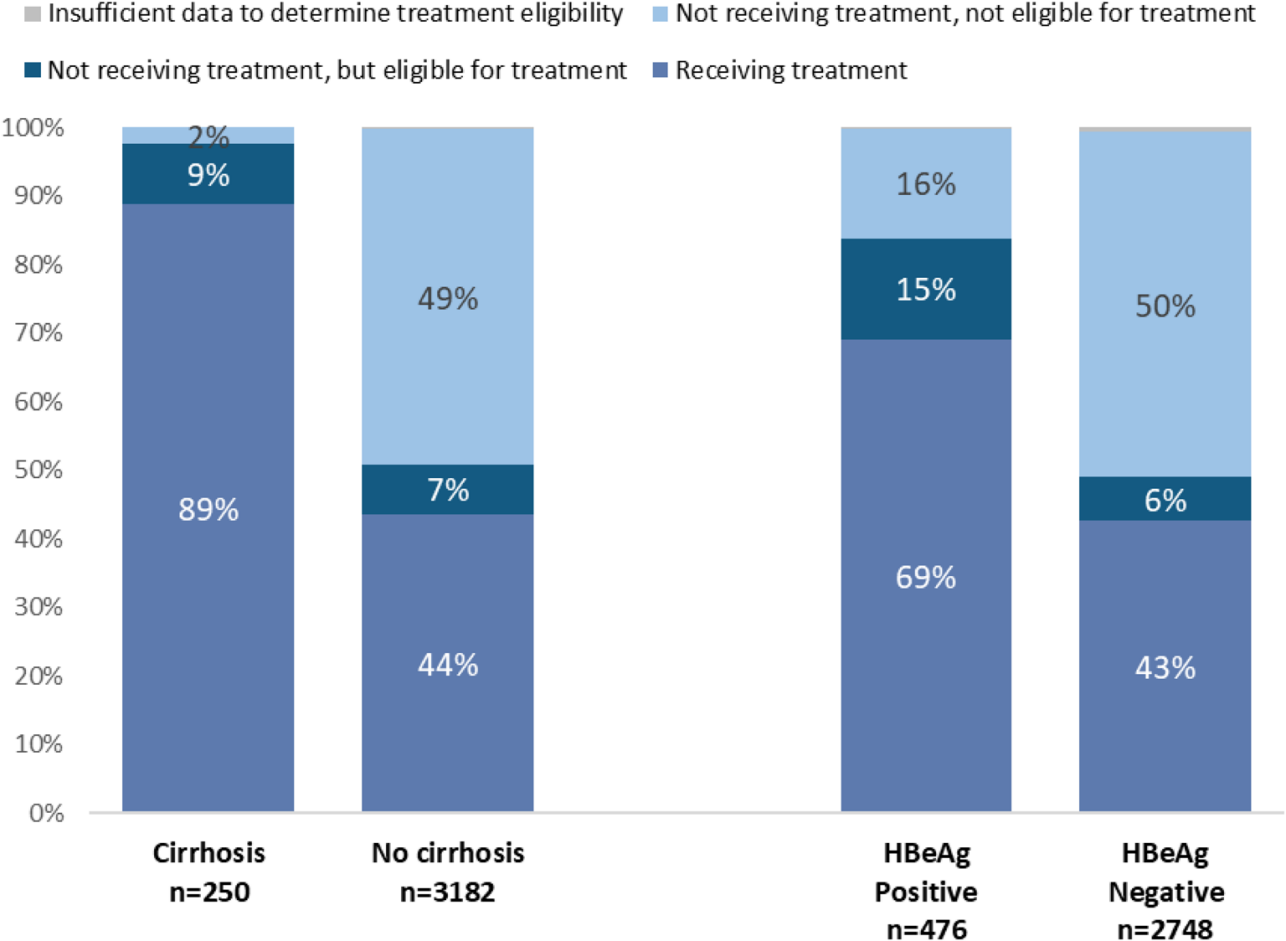
Proportion of participants receiving HBV antiviral therapy and those eligible for treatment based on the PBS criteria, by cirrhosis and HBeAg status.

In logistic regression analysis including total study population, the likelihood of not receiving HBV antiviral therapy despite eligibility (as defined by PBS criteria) was higher among women [adjusted OR (aOR): 1.59, 95% CI: 1.34, 1.88]. Compared with individuals older than 60, younger individuals were more likely to remain untreated despite eligibility, with the highest likelihood observed among those aged 40 years or younger (aOR: 2.65, 95% CI: 1.60, 4.40). A similar trend was observed when the analysis was restricted to participants who were being monitored without treatment, with women and younger individuals more likely to remain untreated despite being eligible (Table 2).

**TABLE 2:** Demographic factors associated with not receiving HBV antiviral therapy despite eligibility.^a^

	All participants with available data (*n* = 3443)		Only participants who were monitored without treatment (*n* = 1816)	
	Adjusted OR (95% CI)	*p*	Adjusted OR (95% CI)	*p*
Sex				
Male	1.00		1.00	
Female	1.59 (1.34, 1.88)	< 0.001	1.31 (1.12, 1.52)	0.001
Age				
> 60 years	1.00		1.00	
41–60 years	1.51 (1.01, 2.28)	0.047	1.29 (0.88, 1.89)	0.191
≤ 40 years	2.65 (1.60, 4.40)	< 0.001	2.09 (1.35, 3.24)	0.001
First Nations Australians				
No	1.00		1.00	
Yes	1.56 (0.58, 4.24)	0.379	0.91 (0.32, 2.53)	0.852
Region of birth				
Australia	1.00		1.00	
East or South‐East Asia	0.84 (0.57, 1.25)	0.397	1.02 (0.54, 1.92)	0.962
Other regions	0.72 (0.43, 1.20)	0.205	0.59 (0.33, 1.05)	0.075
Not available	0.66 (0.12, 3.51)	0.623	0.65 (0.09, 4.43)	0.661

^a^ Participants included in the analysis were those with available data to identify treatment eligibility and data on sex, age, First Nations Australians identity, and region of birth.

## 4 Discussion

The REACH‐B study, a large cohort study of people with chronic HBV infection in Australia, provides a real‐world profile of demographic and clinical characteristics of people engaged with a range of HBV clinical services across the country. Within this selected clinical population, most patients (92%) received recommended HBV clinical care (either antiviral therapy or monitoring) as defined by Australian PBS criteria. A similar proportion was observed among individuals with cirrhosis (91%), while it was lower among those HBeAg positive (85%). Despite high adherence to guideline‐based care, women and younger individuals were more likely to remain untreated while technically eligible by guidelines. When international guidelines with broader treatment eligibility criteria than current Australian guidelines were applied, the proportion of people with chronic HBV infection who could be considered treatment eligible markedly increased.

In Australia, people born overseas, and people who identified as First Nations Australians represent the largest populations living with chronic HBV infection [22]. An estimated 73% of people with chronic HBV infection in Australia are born overseas, primarily in North‐East or South‐East Asia (46%) and Sub‐Saharan Africa (5%), while 7% are First Nations Australians [22]. Consistent with these estimates, 49% of participants in the REACH‐B study were born in North‐East or South‐East Asia and 6% in Sub‐Saharan Africa, while 27% identified as First Nations Australians. These findings suggest that the REACH‐B study adequately represents priority populations for HBV care in Australia, with the higher representation of First Nations Australians due to the inclusion of study sites from clinics serving these populations, particularly in the Northern Territory and Queensland.

The prevalence of HDV co‐infection was 2% among participants with available data while in 66%, data on HDV test results were not available. Despite national and international guidelines recommending routine assessment for HDV in all individuals with HBV infection [3, 4, 5, 6, 7], testing in practice often occurs only when there is a specific clinical indication, particularly in settings where the known prevalence is almost zero, such as among the First Nations Australians [28]. This limited testing also partly reflects restricted access to laboratories with HDV testing capability as well as financial considerations. Consequently, our estimate might not be the true prevalence of HDV co‐infection in Australia, underscoring the need for improved access to diagnostic services and broader implementation of testing guidelines.

In the REACH‐B study population, 47% were receiving antiviral therapy, with another 7%–8% eligible but not receiving therapy, based on both Australian clinical guidelines and the Australian prescribing criteria (PBS). Assuming those people who were receiving antiviral therapy were eligible at the time they initiated treatment, we estimate that 54%–55% of REACH‐B participants were ever eligible for antiviral therapy. This is considerably higher than in a modelling study, which estimated that among the 219,800 individuals living with chronic HBV infection in Australia in 2023, 30% were eligible for treatment [23]. This difference can be explained by the design of the REACH‐B study, a clinic‐based cohort study, comprising participants who were already engaged with clinical services. Given concerns about Australia meeting HBV elimination targets [23], it is important to identify where efforts should be prioritised. These findings suggest that among those engaged in care, guideline‐based management is being implemented effectively. Greater focus is therefore needed on improving diagnosis and linkage to care to reach individuals not yet connected with clinical services.

It should be noted that although PBS criteria for HBV prescribing are largely consistent with the national guideline recommendations on HBV treatment, their purposes are different. The national clinical guidelines are designed to guide best‐practice decision‐making by clinicians and allow for individualised care based on clinicians' clinical judgement. However, the PBS criteria serve a regulatory purpose, defining eligibility for prescribing subsidised treatment under the public program and considering factors beyond clinical benefit, such as cost‐effectiveness and budget impact. Nevertheless, applying the slightly broader Australian clinical guidelines made very little difference—only increasing the proportion eligible for treatment by 1%.

Our findings identified that although the overall proportion of individuals eligible but not receiving antiviral therapy under the PBS criteria was low (7%), it was at a comparable level among those with cirrhosis (9%). People living with cirrhosis are a key population in whom treatment is recommended for any level of detectable HBV DNA. It is not clear why these 22 individuals did not appear to be receiving treatment, but possibilities include recent engagement with services, non‐adherence or other patient‐related factors including personal choice and an individualised risk–benefit analysis. When evaluated by HBeAg status, a higher proportion of participants who were HBeAg positive remained untreated despite eligibility (14% vs. 5% in those HBeAg negative). Our regression model (which could not include HBeAg or other treatment determining factors) also indicated that younger age and female sex were more likely to fall in this category. Taken together, we hypothesise that in young HBeAg positive individuals, especially women in whom the ALT cut‐off is as low as 19 IU/L, other factors, assessed by both clinician and patient, may be determining the decision as to when to start treatment [29]. Some other studies have also reported lower treatment uptake among women and suggested that lower ALT thresholds (compared to men) or social barriers, such as limited health literacy and knowledge, may contribute to this disparity [30, 31, 32]. Gender discrimination in medical care generally is also widely recognised [33], and may be a relevant contributor. Encouragingly, in our analysis of antiviral therapies by age and sex, a marked shift in preferred antiviral use away from entecavir was observed among women, from 68% in the overall treated cohort down to 35% among women of childbearing age (18–44 years). In this group, tenofovir was the preferred antiviral (63%), reflecting the recommendations for tenofovir use in pregnancy and providing reassurance of clinicians' adherence to optimal care.

Other possible explanations for the apparent lack of adherence to treatment eligibility criteria, particularly in younger individuals, may relate to alternative causes of ALT elevation. Lifestyle‐related factors such as alcohol use, obesity, levels of physical activity or metabolic dysfunction, which were not assessed in our study, are common contributors to elevated ALT levels [29]. Steatotic liver disease (SLD), including alcohol‐associated SLD (ALD) and metabolic dysfunction–associated SLD (MASLD), is common among people with HBV, with an estimated prevalence of 36% [34]. In a large HBV cohort with a mean follow‐up of 7.7 years, alcohol consumption and non‐alcoholic fatty liver disease accounted for 31% and 25% of ALT elevations, respectively [35]. Among 236 First Nations Australians with HBV infection who participated in a study, 60% had obesity and 24% were drinking alcohol hazardously [29]. Interestingly, in our current study, when using an ALT threshold of 19 IU/L for women and 30 IU/L for men, as recommended by most guidelines, 55% of the REACH‐B participants with undetectable or unquantifiable HBV DNA had an elevated ALT, suggesting a high likelihood of other causes of elevated ALT in this population. It is thus possible that treating clinicians, with optimal insight into individual patient circumstances, considered alternative causes of ALT elevation more probable in some patients and elected not to commence HBV antiviral therapy despite HBV DNA and ALT levels meeting treatment eligibility criteria. Clinicians, and patients, may have been particularly cautious about initiating long‐term antiviral therapy in younger individuals [36]. Further, given that this study represents a single cross‐sectional assessment, it is possible that younger ‘eligible’ patients were more likely to undergo a short period of observation (e.g., 3–6 months) to allow for a possible spontaneous transition to immune control before treatment initiation. Longitudinal analysis of the REACH‐B cohort will further elucidate this.

In recent years, many guidelines have adopted broader treatment eligibility criteria, partly to address suboptimal HBV testing and treatment rates, particularly in resource‐limited settings. In 2024, the WHO released new HBV clinical guidelines that prioritise expanded and simplified treatment eligibility criteria to facilitate treatment scale‐up [37]. In our study, treatment eligibility among those under monitoring increased from 14% under PBS criteria to 25% when applying WHO recommendations. Similarly, other studies have reported a substantial increase in treatment eligibility using WHO criteria [18, 19, 20, 21].

Given the considerable variability among current HBV treatment guidelines, the proportion of our study participants who are currently untreated but potentially eligible varies from as low as 3% (APASL 2015) to as high as 83% (Chinese Medical Association 2022). These differences largely reflect different target audiences and priorities across the guidelines, as well as the evolving nature of the HBV treatment discussion [38]. The WHO promotes expanding HBV care access through decentralised models, including task‐shifting to non‐specialists [37], with greater focus on targeting health care providers in low‐ and middle‐income countries. International liver disease societies, primarily focused on high‐income countries with easier access to specialist services and resources, may reflect more targeted approaches [39]. There have been some successful experiences of using simplified HBV guidelines to address challenges in access to specialist services and costly tests in low‐ and middle‐income countries [40, 41]. Recent advances in point‐of‐care HBV DNA testing, including fingerstick‐based testing, also have the potential to facilitate decentralised HBV care and the implementation of simplified HBV guidelines [15]. Further studies are needed to evaluate the extent to which the WHO guidelines are implemented in practice and also to assess how simplifying treatment eligibility criteria impacts treatment uptake and outcomes, as well as whether the widened eligibility aligns with patients' preferences. Recent advances in point‐of‐care HBV DNA testing, including fingerstick‐based testing,potential role in facilitating decentralised HBV care.

This study has several limitations. The REACH‐B study population includes individuals who attended and were managed in services participating in a national HBV research collaboration, not fully representative of the overall population living with chronic HBV infection in Australia. Approximately 16% of participants were excluded from the analysis given unavailable data on HBV DNA and ALT. Given the large number of study sites and study population, the data collection process was intentionally limited to variables routinely collected in participating clinics to align with standard clinical practice and reduce site burden. Despite this, missing data were unavoidable given the reliance on existing clinical records. We evaluated treatment eligibility based on cross‐sectional data of ALT and HBV DNA levels although these levels fluctuate over time. Therefore, individuals with values near the eligibility threshold may shift status in subsequent visits. Moreover, we could not evaluate treatment eligibility among participants already receiving treatment. Co‐morbidities and family history of cirrhosis or HCC were not considered in the treatment eligibility criteria given unavailable data for many participants.

In conclusion, the REACH‐B study provides important data on a large cohort of people with chronic HBV infection in Australia that helps inform resource allocation and improving access to care. Reassuringly among REACH‐B participants, who are all engaged in HBV services, most eligible individuals based on the PBS criteria receive treatment. However, this may not be the case outside of these settings. Modelling and surveillance data suggest that only half of all people estimated to be eligible for HBV antiviral therapy in Australia are currently receiving it [23], likely reflecting large gaps in diagnosis and overall linkage to care. Using alternative guideline recommendations for antiviral therapy, the population eligible for treatment will increase by anywhere between 13% and 44%. These data provide important insights into the potential impact of expanding treatment eligibility. Lower treatment uptake among women and younger people may partly reflect the lower threshold for elevated ALT in women, or limitations of PBS criteria for more nuanced treatment decision‐making in younger people where elevated ALT may be attributed to non‐HBV factors. As treatment guidelines evolve and eligibility criteria broaden, it will be essential to monitor these patterns and the associated outcomes while also incorporating the perspectives of communities and people with lived experience.

## Funding

The Kirby Institute and the Doherty Institute are funded by the Australian Government Department of Health, Disability and Ageing. The REACH‐B study has been funded by the Department of Health, Disability and Ageing (GO5055). B.H. (2026968), M.M. (2034418), G.J.D. (2008276) and G.V.M. (2016698) are supported by National Health and Medical Research Council of Australia (NHMRC) Investigator Grants. J.G. is supported by the Robert W. Storr Bequest to the Sydney Medical Foundation, The University of Sydney; an NHMRC Program Grants (APP1053206, APP1149976), Investigator and MRFF grants (GNT2032407; NCRI000183; GNT2016215; GNT2010795; GNT1196492) and a Cancer Institute NSW grant (2021/ATRG2028). T.T. is supported by the Paul and Valeria Ainsworth Precision Medicine Fellowship; the GESA 2025 Lawrie Powell AC Mid‐Career Grant; NHMRC Ideas grant (APP2002565; APP20021586) and the Australian Centre for HIV and Hepatitis Virology Research.

This research was produced in part by UNSW Sydney researchers and is subject to the UNSW Intellectual property policy. For the purposes of Open Access, the author has applied a Creative Commons Attribution CC BY licence to any Author Accepted Manuscript (AAM) version arising from this submission.

## Ethics Statement

This study is conducted in accordance with the ethical principles laid out in the Declaration of Helsinki and the National Statement on Ethical Conduct in Research Involving Humans. The study protocol was approved by St. Vincent's Hospital Sydney Human Research Ethics Committee (2021/ETH01134). As a clinical audit study, a waiver of individual consent was granted.

## Conflicts of Interest

G.V.M. reported grants from ViiV and Janssen, received honoraria from ViiV and Gilead, and participated on a Data Safety Monitoring Board for ViiV. G.J.D. declares clinical trial grants from Gilead Sciences and AbbVie. J.G. has served on advisory boards or has received speaker fees from Roche, Astra Zeneca, Novo Nordisk, BMS, Pfizer, Cincera, Pharmaxis, Boehringer Mannheim, CSL, Norgine, Gilead, Eisai and Ipsen. M.M. has participated in advisory boards for Abbvie. A.J.W. has received funding for research to her institution from Gilead, AbbVie and Cepheid separate to the current work. R.N. received honoraria from Gilead and participated in, and declares trial grants from Gilead. J.O. has received speaker fees or served on advisory boards for Novo‐nordisk, Ipsen, Roche diagnostics, Norgine and Gilead. T.T. serves as consultant to Excision BioTherapeutics and Kerna Ventures with fees to his institution, as paid consultant to Gilead Sciences and GSK, and received payment for speaking engagements from GSK, ASHM and the Singapore HBV Cure conference. The other authors declare no conflicts of interest.

## Supporting information


**Supplementary Figure 1:** Simplified eligibility criteria for anti‐viral therapy in chronic HBV infection, based on various clinical guidelines. Co‐morbidities and extrahepatic manifestations have not been included.
**Supplementary Table 1:** The HBV treatment criteria specified by the Pharmaceutical Benefits Scheme (PBS) via the S100 Highly Specialised Drugs scheme.
**Supplementary Table 2:** Baseline characteristics of participants at enrolment, by study site.

## Data Availability

The data that support the findings of this study are available on request from the corresponding author. The data are not publicly available due to privacy or ethical restrictions.
