# Supplementary material for: Anti‐Viral Treatment Eligibility and Treatment Uptake Among People With Chronic Hepatitis B in Australia: The REACH‐B Study
Source: J Viral Hepat. 2026 Nov 2;33(12):e70238. doi: 10.1111/jvh.70238 (PMC13726446; doi:10.1111/jvh.70238)
Supplement: Supplementary file 1 [file JVH-33-0-s001.docx]

**SUPPLEMENTARY MATERIALS**

**Supplementary Figure 1:** Simplified eligibility criteria for anti-viral therapy in chronic HBV infection, based on various clinical guidelines. Co-morbidities and extrahepatic manifestations have not been included.

a. Including individuals with advanced liver disease (liver fibrosis score ≥F3)
b. HBV DNA > 2000 IU/mL for compensated cirrhosis and any detectable HBV DNA for decompensated cirrhosis
c. HBeAg positive: Liver fibrosis score ≥F2 and elevated ALT or liver fibrosis score ≥F2 and HBV DNA>6 log; HBeAg negative: Liver fibrosis score ≥F3
d. Liver fibrosis score ≥F2 and elevated ALT.
e. EASL and AASLD guidelines recommend treatment in the presence of risk factors for HCC

**Supplementary Table 1:** The HBV treatment criteria specified by the Pharmaceutical Benefits Scheme (PBS) via the S100 Highly Specialised Drugs scheme

**Individuals with cirrhosis**

***Clinical criteria:***

- Patient must have cirrhosis,

**AND**

- Patient must have detectable HBV DNA.

**Individuals with cirrhosis**

***Clinical criteria:***

- Patient must not have cirrhosis,

**AND**

- Patient must have elevated HBV DNA levels greater than 20,000 IU/mL (100,000 copies/mL) if HBeAg positive, in conjunction with documented hepatitis B infection; **OR**
- Patient must have elevated HBV DNA levels greater than 2,000 IU/mL (10,000 copies/mL) if HBeAg negative, in conjunction with documented hepatitis B infection,

**AND**

- Patient must have evidence of chronic liver injury determined by confirmed elevated serum ALT or liver biopsy.

**Supplementary Table 2:** Baseline characteristics of participants at enrolment, by study site

| **Baseline characteristics** | **St Vincent Hospital, NSW  n=231** | **Westmead Hospital, NSW n=624** | **Blacktown Hospital, NSW n=137** | **Justice Health, NSW n=211** | **Barwon Health, Vic n=92** | **Utopia Refugee Health, Vic n=131** | **Cairns Hospital, Qld n=351** | **Torres & Cape Hospital, Qld n=349** | **Sunshine Coast Hospital, Qld n=90** | **Royal Adelaide Hospital, SA n=450** | **Queen Elizabeth Hospital, SA n=507** | **Kimberley Health Services, WA n=23** | **Goldfields Health Services, WA n=25** | **Great Southern Health Services, WA n=11** | **Alice Spring Hospital, NT n=36** | **Royal Darwin Hospital, NT n=349** | **Top End Medical Centre, NT n=31** |
| --- | --- | --- | --- | --- | --- | --- | --- | --- | --- | --- | --- | --- | --- | --- | --- | --- | --- |
| **Female, n (%)** | 88 (38) | 289 (46) | 71 (52) | 18 (9) | 42 (46) | 53 (41) | 178 (51) | 174 (50) | 69 (77) | 193 (43) | 240 (47) | 11 (48) | 11 (44) | 5 (45) | 16 (44) | 135 (39) | 17 (55) |
| **Median age** | 49 | 51 | 48 | 44 | 50 | 47 | 45 | 50 | 54 | 48 | 49 | 56 | 46 | 57 | 48 | 46 | 50 |
| **First Nations Australians, n (%)** | 1 (<1) | 2 (<1) | 0 | 84 (40) | 2 (2) | 0 | 179 (51) | 333 (95) | 2 (2) | 2 (<1) | 6 (1) | 17 (74) | 6 (24) | 0 | 36 (100) | 333 (95) | 0 |
| **Born in Australia, n (%)** | 32 (14) | 15 (2) | 7 (5) | 107 (51) | 14 (15) | 0 | 197 (56) | 317 (91) | 12 (13) | 35 (8) | 26 (5) | 18 (78) | 7 (28) | 0 | 36 (100) | 334 (96) | 0 |
| **HBeAg positive, n (%)** | 50 (22) | 118 (19) | 24 (18) | 29 (14) | 9 (10) | 17 (13) | 33 (9) | 52 (15) | 13 (14) | 42 (9) | 65 (13) | 2 (9) | 2 (8) | 1 (9) | 1 (3) | 40 (11) | 1 (3) |
| **Cirrhosis, n (%)** | 26 (11) | 41 (7) | 1 (<1) | 17 (8) | 17 (18) | 11 (8) | 24 (7) | 18 (5) | 7 (8) | 27 (6) | 20 (4) | 5 (22) | 1 (4) | 3 (28) | 1 (3) | 34 (10) | 2 (6) |
| **Receiving anti-viral treatment, n (%)** | 135 (58) | 461 (74) | 39 (28) | 77 (36) | 86 (92) | 66 (50) | 97 (28) | 50 (14) | 43 (52) | 244 (54) | 196 (39) | 18 (78) | 6 (24) | 7 (73) | 4 (11) | 113 (32) | 20 (65) |
